# A Serum Biomarker Panel of exomiR-451a, exomiR-25-3p and Soluble TWEAK for Early Diagnosis of Rheumatoid Arthritis

**DOI:** 10.3389/fimmu.2021.790880

**Published:** 2021-11-15

**Authors:** Samantha Rodríguez-Muguruza, Antonio Altuna-Coy, Sonia Castro-Oreiro, Maria José Poveda-Elices, Ramon Fontova-Garrofé, Matilde R. Chacón

**Affiliations:** ^1^ Disease Biomarkers and Molecular Mechanisms Group, Institut D'investigaciò Sanitària Pere Virgili (IISPV), Universitat Rovira i Virgili, Tarragona, Spain; ^2^ Rheumatology Section, Joan XXIII University Hospital, Tarragona, Spain

**Keywords:** early rheumatoid arthritis, sTWEAK, sCD163, exomiRNAs, exosomes

## Abstract

**Background:**

The etiology of rheumatoid arthritis (RA) remains poorly understood. Early and accurate diagnosis still difficult to achieve. Inflammatory related molecules released into the circulation such cytokines and exosome-derived microRNAs (exomiRNAs) could be good candidates for early diagnosis of autoimmune diseases. We sought to discover a serum biomarker panel for the early detection of RA based on exomiRNAs and inflammatory markers.

**Methods:**

A 179 miRNAs-microarray panel was analyzed in a pilot study (4 early RA and 4 controls). Validation of deregulated exomiRNAs was performed in a larger cohort (24 patients with early RA and 24 controls). miRNet software was used to predict exomiRNA gene-targets interactions. Potentially altered pathways were analyzed by Reactome pathway database search. STRING database was used to predict protein-protein interaction networks. Enzyme-linked immunosorbent assay was used to measure serum levels of sTWEAK and sCD163. Signature biomarker candidates were statistical analyzed.

**Results:**

We detected 11 differentially expressed exomiRNAs in early RA pilot study. Validation analysis revealed that 6/11 exomiRNAs showed strong agreement with the pilot microarray data (exomiR-144-3p, -25-3p, -15a-5p, -451a, -107 and -185-5p). sTWEAK and sCD163 biomarkers were significantly elevated in the serum of patients with early RA. Receiver operating characteristic (ROC) analysis showed that the best panel to diagnose early RA contained exomiR-451a, exomiR-25-3p and sTWEAK, and could correctly classify 95.6% of patients, with an area under the ROC curve of 0.983 and with 100% specificity and 85.7% sensitivity. The *YWHAB* gene was identified as a common target of the putative miRNA-regulated pathways.

**Conclusion:**

A novel serum biomarker panel composed of exomiR-451a, exomiR-25-3p and serum levels of sTWEAK may have use in the early clinical diagnosis of RA. A new predicted exomiRNA-target gene *YHWAB* has been identified and may have a relevant role in the development of RA.

## Introduction

Rheumatoid arthritis (RA) is a chronic autoimmune disease characterized by inflammation and degradation of the peripheral joints (1). The pathophysiology of RA is complex, and its etiology is unknown, which limits successful treatment options. Given the heterogeneity in clinical RA manifestations and the variability of therapeutic response (2), the identification of early stage disease is essential to halt/slow its evolution.

Currently, the American College of Rheumatology (ACR)/European League Against Rheumatism EULAR 2010 use rheumatoid factor (RF) and/or antibodies against cyclic citrullinated proteins (ACPA) (3, 4) for RA diagnosis. Other diagnostic autoantibodies have been reported that can help in the early diagnosis of RA, including autoantibodies to vimentin (5), carbamylated proteins (6), and levels of 14-3-3 η protein (YWHAH) (7). However, biomarkers capable of guiding early detection for personalized treatment are urgently needed in RA.

Synovial fibroblasts and activated immune cells are responsible for the production of a large number of inflammatory cytokines, which are believed to play crucial roles in the development and progression of RA. Accordingly, the measurement of cytokines in synovial fluids and serum have been investigated to predict clinical disease activity (8–10). Tumor necrosis factor-like weak inducer of apoptosis (TWEAK) is an inflammatory cytokine (11) produced by several cells of the immune system (natural killer cells and macrophages, among others) (12). TWEAK can be found on cell membrane surface (mTWEAK) and can also be released as a soluble form (sTWEAK) through proteolytic processing; both forms bind to its signal transducer receptor Fn14 (13). sTWEAK has been proposed as a mediator of inflammation and bone erosion in RA, with an active role in synovial fibroblast proliferation and production of inflammatory cytokines (14). High serum levels of sTWEAK have been reported in patients with RA (15) and correlate with disease activity scores. TWEAK can also bind to the CD163 receptor (cluster of differentiation 163), which acts as a scavenger receptor since binding does not transduce intracellular signals, but rather mediates the elimination of sTWEAK, contributing to its degradation (16). Similar to TWEAK, the transmembrane scavenger receptor CD163, which is exclusively expressed by the monocyte–macrophage cell lineage, can be proteolytically detached and shed into circulation as a soluble form (sCD163) (17). Elevated serum levels of sCD163 have been reported in patients with long-standing active RA (18), and it has also been proposed as a marker of disease activity and progression in early RA (19).

Recent evidence has indicated that secreted extracellular vesicles termed “exosomes”, which are involved in intercellular communication by packaging and shuttling various cargo molecules including microRNAs (miRNAs) to recipient cells, may play a critical role in autoimmune-related pathologies (20, 21). Moreover, the miRNA cargo of exosomes (exomiRNAs) has potential diagnostic value as biomarkers in several autoimmune diseases (22). Exosomes and their miRNA cargo have been isolated from different types of bodily fluids in patients with RA (23). For instance, several serum exomiRNAs have been found to be associated with RA disease activity (exomiR-1915-3p, -6511b-5p) (24) and have utility as diagnostic tools (exomiR151a-3p, -199a-5p, -370-3p, -589-5p, -769-5p) (25). The serum exomiRNA miR-548a, which has a role in cell proliferation by regulating TLR4/NF-ĸB signaling, has been reported to be deregulated in patients with RA (26). ExomiRNAs have also been isolated from fibroblast-like synoviocyte conditioned medium in cell models after stimulation with TNFα (exomiR-155-5p, -146a-5p, -323a-5p, -1307-3p) (27) and from synovial fibroblast in a mouse model of RA (exomiR-221) (28).

Despite the known participation of several exomiRNAs in RA, much less is known about their potential utility is diagnosing early disease. A recent publication in a small cohort of 5 patients with early RA identified exomiR-361-5p as a potential biomarker of early disease onset (29). Therefore, we aimed to identify a panel of exomiRNA candidates that, in conjunction with other serum biomarkers, could help clinicians in the early diagnosis of RA. We performed microarray profiling of exomiRNAs isolated from serum exosomes in a well-characterized and representative cohort of patients with early RA *versus* a matched control group. Putative targets of selected exomiRNAs and signaling pathways were also analyzed to identify new therapeutic targets.

## Methods

### Patients and Study Design

Patients (n=28) were classified as having early RA by certified clinical rheumatologists according to ACR/EULAR 2010 criteria (30), with symptoms duration <6 months and not receiving treatment with glucocorticoids or synthetic or biological disease-modifying drugs. Control subjects (n=28) were healthy volunteers with no relevant medical history. Groups were sex and age matched. All patients were recruited from the outpatient clinic of the Rheumatology Service at the University Hospital Joan XXIII, Tarragona, Spain. The study was performed according to the provisions of the Declaration of Helsinki, was approved by the local ethics committee, and adhered to current legal regulations (Bio-medical Research Law 14/2007, Royal Decree of Biobanks 1716/2011, Organic Law15/1999 of September 13 Protection of Personal Data). All methods were approved by the Ethical Committee for Clinical research (CEIM) of the Pere Virgili Research Institute (Ref. CEIM: 047/2021). All participants gave written informed consent. The following exclusion criteria were applied: patients unwilling or unable to provide informed consent; patients having a diagnosis of any systemic inflammatory condition other than RA, such as (but not limited to) juvenile chronic arthritis, spondyloarthropathy, Crohn’s disease, ulcerative colitis, psoriatic arthritis, active vasculitis, or gout (participants with secondary Sjogren’s syndrome were not excluded); history or presence of cardiovascular, respiratory, hepatic, gastrointestinal, endocrine, hematological, neurological, or neuropsychiatric disorders or any other serious and/or unstable illness that, in the opinion of the investigator, could constitute a risk when taking investigational samples or could interfere with the interpretation of data.

### Analytical Methods

Fasted blood was extracted and glucose, cholesterol, triglyceride, high-density lipoprotein cholesterol, Gamma Glutamyltransferase and creatinine levels was performed as described (31). Levels of sTWEAK and sCD163 in serum were determined in duplicate using commercially available human enzyme-linked immunosorbent assay (ELISA) DuoSet Kits (R&D Systems Europe, Abingdon, UK). C-reactive protein and ACPA peptide levels were determined using standard enzymatic methods.

### Exosome Analysis

Exosomes from 300 μl of serum from an early RA patient were isolated using a procedure modified from the exoEasy Serum/Plasma Midi Kit (Qiagen, BioNova Cientifica, Madrid, Spain), by eluting exosomes in 500 μl of elution buffer before extracting miRNA-cargo. Eluates containing intact exosomes were concentrated using 100,000 Da cut-off concentrator (Amicon Ultra-0.5 ml Centrifugal Filters, Millipore). Concentrated exosomes were ultrasonicated and total protein was quantified with BCA method (Pierce). Ten μg of exosome protein and 10 μg human monocytic cell line extract (THP-1), used as whole cell extract control, was loaded on 4–15% SDS-PAGE gels and immunoblotted with polyclonal rabbit antibodies against: EXOAB-CD9A1, EXOAB-CD81A-1, EXOAB-CD63A-1, EXOABHsp70A-1, EXOAB-TSG101-1 (System Biology, Palo Alto, CA, USA). An antibody to tubulin (Thermo Fisher, Rockford, IL) was used as a loading control (Thermo Fisher, Rockford, IL, USA). HRP-conjugated goat anti-mouse or anti-rabbit (both from SBI) were used as secondary antibodies. Signal detections were developed with Super Signal West Femto chemiluminescent substrate (Pierce Biotechnology, Boston, MA, USA) and capture by VersaDoc imaging system and Quantity One software (Bio-Rad, Barcelona, Spain).

### Transmission Electron Microscopy

Isolated exosomes were placed on carbon-coated copper grids (200 mesh) and incubated in osmium tetroxide vapor for 15–30 min. Images were collected using a JEOL 1011 transmission electron microscope (Jeol, Tokyo, Japan) operating at 80 kV with a megaview III camera (Olympus Soft Imaging Solutions GmbH, Munster, Germany).

### ExomiRNA Expression Profiling Using TaqMan Low-Density Arrays

ExomiRNAs were extracted from 300 μl of serum using the exoRNeasy Serum/Plasma Midi Kit (Qiagen, BioNova Cientifica, Madrid, Spain). We initially performed a pilot study using 8 randomly selected subjects (4 with early RA and 4 controls). For exomiRNA screening, the miRCURY LNA Universal RT microRNA PCR, Polyadenylation and cDNA Synthesis Kit (Qiagen, BioNova Cientifica, s.l. Madrid, Spain) was used for reverse transcription. cDNA was diluted and assayed by qRT-PCR according to the user’s protocol in a 7900HT Fast Real-Time PCR System (Applied Biosystems, Foster City, CA, USA). Each sample was assayed using ExiLENT SYBR Green Master Mix on a miRCURY LNA miRNA Human Serum/Plasma Focus PCR Panel of 179 highly expressed human miRNAs in serum/plasma (Qiagen). Fluorescence readings and expression records of the exomiRNAs during the qRT-PCR were performed with the SDS 2.3 program (Applied Biosystems). Analysis of raw microarray qRT-PCR data was performed by Geneglobe Data Analysis Software (https://geneglobe.qiagen.com/us/analyze). This software normalizes the data by using the UniSp3 miRNA values to eliminate inter-microarray plate differences. A cycle threshold (C_T_) cut off <35 was applied, since C_T_ values >35 are categorized by the software as inconclusive data. C_T_ values for each sample were normalized to the arithmetic mean of 4 selected miRNAS that showed no differences between studied groups: hsa-miR-423-5p, has-miR-374b-5p, has-miR-181a-5p, has-miR-150-5p and, the resulting value is known as ΔC_T_. A calibrator (a sample made by mixing 4 patients, 2 early RA and 2 controls) were included in the plates for the purpose of comparison between groups, thus each miRNA regardless of the condition was normalized to the ΔC_T_ of the calibrator sample (ΔΔCT = ΔCT sample -ΔCT calibrator). The fold change expression of each exomiRNA was calculated with the formula 2^-ΔΔCT^. ExomiRNAs with p ≤ 0·05 and expression values ≥ 1.8-fold or ≤ -1.8-fold were considered for further validation analysis. Selected microarray exomiRNAs that accomplished the above criteria were validated in n=24 early RA samples, n=24 controls and again in pilot samples by using individual probes purchased to Qiagen **(raw C_T_ data provided in** **Supplementary Material**).

### Target Analysis and Pathway and Functional Enrichment

miRNet (https://www.mirnet.ca) was used to predict exomiRNA targets. Potentially altered pathways were analyzed with Reactome (https://reactome.org) in miRNet software using a hypergeometric test algorithm. Enriched pathways with a p-value < 0.05 and enrichment score > 1 are expected to contain over-represented exomiRNA targets. The STRING database (https://string-db.org) was used to predict protein-protein interaction networks and to perform functional enrichment analysis. Interaction STRING Scores between 0.9 and 1 were considered true by the sorfware (29).

### Statistical Analysis

For the pilot microarray study, sample size was calculated following the Mdanderson bioinformatic software (https://bioinformatics.mdanderson.org/MicroarraySampleSize/). Briefly, measurement of 179 genes per duplicate, considering 50 false positives, 2-fold-change differences between sample groups, a standard deviation of 0.5 and, an estimated power analysis of 0.9, the minimum sample size required was calculated to be 4 patients in each group.

For validation analysis, sample size was calculated using G*Power 3.1.9.7. Assuming a change of 2-fold between groups and similar group variances, with an average power > 90% and a false discovery rate of 5%, a minimum of 19 patients was calculated to be needed in each group. The normality of the anthropometric and clinical variables was analyzed with the Shapiro-Wilk test. The data is shown as median with interquartile range. The non-parametric Mann-Whitney U-test was used to analyze differences in anthropometric, clinical data, biomarkers serum levels (sTWEAK and sCD163) and absolute expression levels of exomiRNAs candidates between studied cohorts. A p-value < 0.05 was considered significant. The strength of the association between variables was calculated using the Spearman correlation test (nonparametric variables). Partial Least Square Discriminant Analysis (PLS-DA), variable importance in projection (VIP) analysis, and univariate logistic regression models’ analysis were performed for selected variables in order to determine the best discriminative model between the groups. Receiver operating characteristic (ROC) analysis was conducted to evaluate the best predictive model. The statistical software SPSS Statistics 24.0 (IBM, Madrid, Spain) package and R software (http://cran.r-project.org) were used for analysis. GraphPad Prism v7 (GraphPad Software Inc., San Diego, CA) was used to plot data results.

## Results

### Characterization of Serum Exosomes

**Figure 1:**
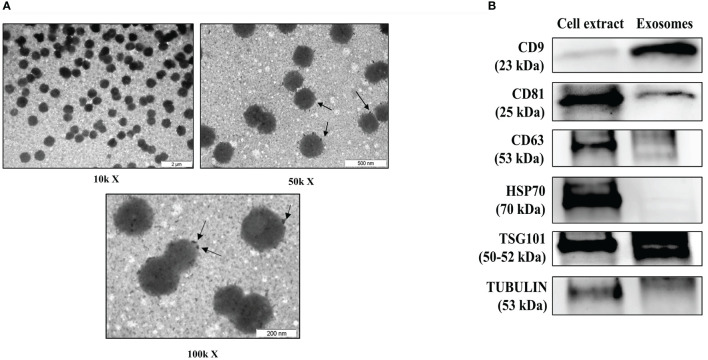
Characterization of serum-derived exosomes. **(A)** Analysis of early RA serum exosomes by electron microscopy. Arrows indicates osmium tetroxide deposition on exosome lipid surface. **(B)** Representative western blot of protein extracts prepared from exosomes isolated from serum of patients with early RA and total cell extract from the human monocytic cell line THP-1 as a control, tested with the following antibodies: CD9, CD81, CD63, HSP70, TSG101 and tubulin. μm, micrometers, kX magnification levels; kDa, kilodaltons.

Isolated serum exosomes from an early RA patient sample was treated with osmium tetroxide to stain lipid-bilayer (32) to test the purity and size by transmission electron microscopy (TEM). TEM osmium staining images revealed different types of dense bodies within 30–150 nm, corresponding to exosomes expected size range (33) (**Figure 1A**). In order to comply with the guidelines of the International Society of Extracellular Vesicles (34), we extracted protein from isolated exosomes and from monocytic cell line THP-1, as a whole cell protein control and, tested for the presence of selected exosome markers by using western blot analysis. We detected CD81, TSG101 and CD63 markers and a clear enrichment on CD9 marker is observed in serum exosome sample (**Figure 1B**). Detailed information about the Western blotting can be found in **Supplementary Material**.

### Serum exomiRNA Microarray Analysis Reveals a Unique Profile in Patients With Early RA

**Figure 2:**
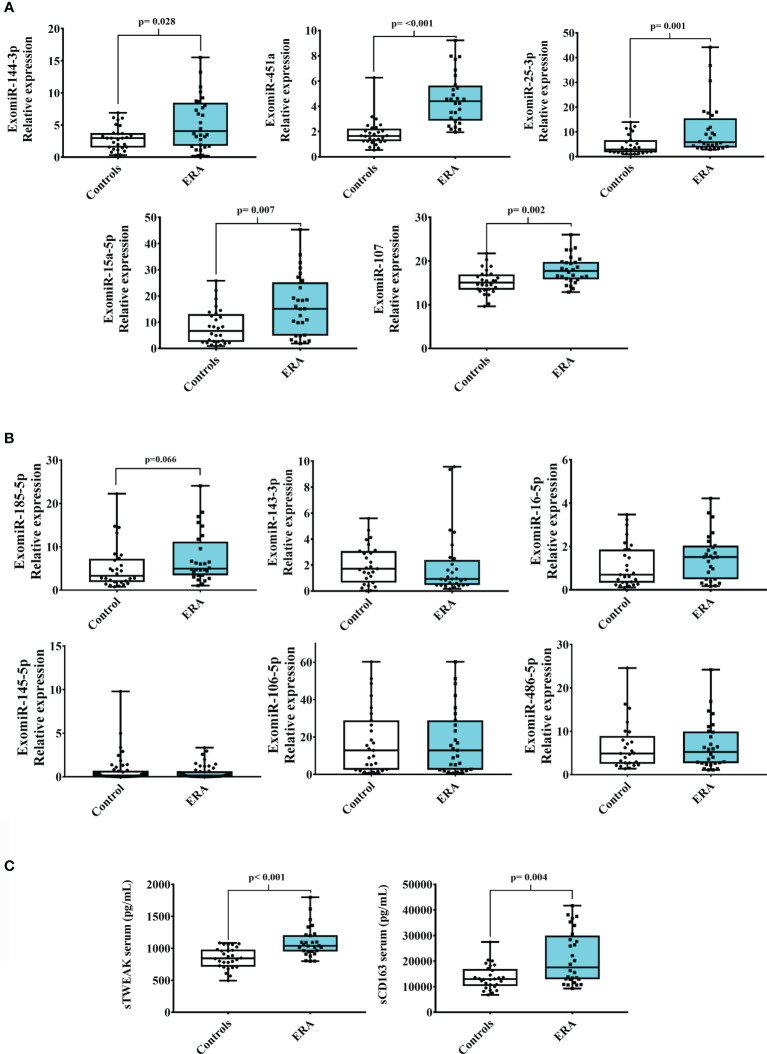
Serum exomiRNAs and inflammatory biomarkers in early RA. Validation of 11 selected exomiRNAs from the microarray pilot study of ERA (early RA) and control cohorts. **(A)** Significant upregulated exomiRNAs **(B)** Not-significantly expressed exomiRNAs. **(C)** sTWEAK and sCD163 serum circulating levels in patients with ERA and controls. Box plots show the median, quartiles and extreme values.

We searched for a useful and practicable biomarker panel for the early diagnosis of RA. The panel search was divided into two phases: the initial pilot phase was established to isolate serum exomiRNAS from 8 participants (4 with early RA and 4 controls), matched by age and gender (**Table S1**). For that we used a microarray profiling microRNA panel that contained 179 miRNAs. Results showed that 11 exomiRNAs were differentially expressed when comparing early RA and control subjects: exomiR-144-3p, -185-5p, -25-3p, -15a-5p, -451a, -107, -143-3p, -16-5p, -145-5p, -106-5p and -486-5p. Then, the expression of the 11 deregulated exomiRNAs were validated in a larger sample (n=24 patients with early RA and n=24 healthy controls), and again in pilot samples (**Table 1**). Validation analysis showed that from the 11-microarray selected exomiRNAs, 5 were still significantly upregulated in early RA patient’s cohort when compared with controls (exomiR-144-3p, -451a, -25-3p, -15a-5p and -107) (**Figure 2A**). ExomiR-185-5p was also upregulated in serum samples of early RA but did not reach significance (**Figure 2B**). The expression of the remaining 5 exomiRNAs (exomiR -143-3p, -16-5p, -145-5p, -106-5p and -486-5p) did not showed significantly differences between early RA and controls (**Figure 2B**).

**Table 1:** Patients’ characteristics.

	Control (n=28)	Early AR (n=28)	p -value
	Median (Range)	Median (Range)	
**Age (Years)**	47.00 (42.00, 53.50)	50.00 (40.50, 58.00)	0.501
**BMI (kg/m^2^)**	27.03 (25.63, 28.89)	27.36 (23.73, 31.11)	1.000
**Glucose (mmol/L)**	80.72 (72.38, 89.98)	86.50 (80.25, 92.75)	0.790
**Uric Acid (mmol/L)**	3.98 (3.13, 4.53)	4.12 (3.37, 5.29)	0.245
**Creatinine (µmol/Ll)**	0.66 (0.61, 0.75)	0.64 (0.58, 0.81)	0.731
**Total Cholesterol (mmol/L)**	191.89 (162.17, 212.74)	185.50 (150.69,201.90)	0.334
**HDL-Cholesterol (mmol/L)**	48.07 (35.23, 55.21)	43.50 (35.00, 51.00)	0.354
**Triglycerides (mmol/L)**	93.81 (55.76, 116.37)	97.00 871.50, 124.5)	0.498
**GGT (µkat/L)**	13.80 (9.75, 22.20)	16.00 (12.00, 23.25)	0.225
**CRP (mg/L)**	0.69 (0.18, 1.14)	9.00 (4.00, 24.00)	<0.001
**ACPA (U/mL)**	1.46 (1.16, 1.61)	124.50 (32.35, 355.25)	<0.001
**RF (UI/ml)**	ND	158.50 (41.25, 297.25)	–

IQ, interquartile, BMI, Body mass index; HDL, High-density lipoprotein; GGT, Gamma Glutamyltransferase; CRP, C-reactive protein, ACPA, anti-citrullinated protein/peptide antibody; RF, Rheumatoid Factor; ND, Not Determined.

### sTWEAK and sCD163 Serum Levels Are Upregulated in Patients With Early RA

Additional biomarkers were also measured in serum samples of early RA patients and controls. We choose to measure serum levels of sTWEAK, an inflammatory cytokine already known to be deregulated in stablish RA patients (15). Also levels of sCD163 were measured in early RA since have been found associated with RA disease activity (19). Results showed that both inflammatory biomarkers were significantly elevated in early RA when compared with controls counterparts (**Figure 2C**).

### miRNA-Target Regulatory Gene and Protein Network Analysis

**Figure 3:**
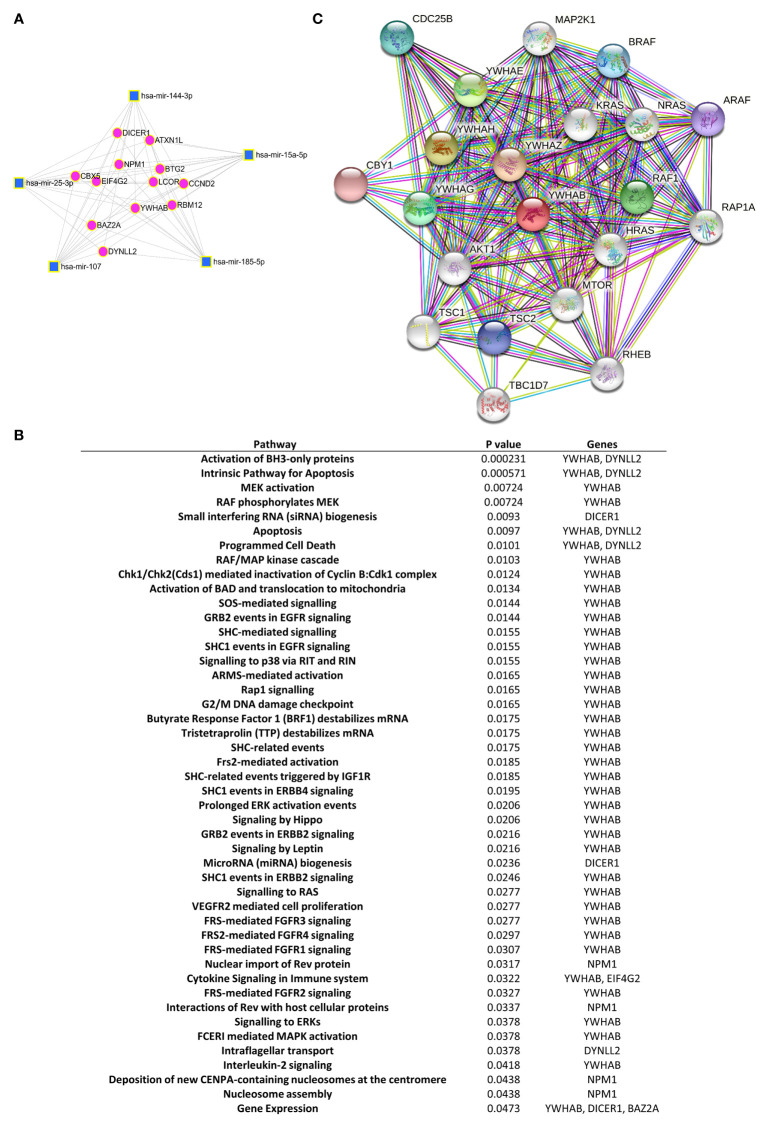
Gene and protein pathway regulatory networks of the selected exomiRNA-targets. **(A)** Network miRNet analysis shows the major set of significantly dysregulated exomiRNAs in the early RA group and their shared target genes. Cluster hubs: squares indicate exomiRNAs and circles indicate their target genes. **(B)** Reactome pathway analysis displays the significant pathways connecting the target genes of the dysregulated exomiRNAs in patients with early RA. **(C)** STRING analysis of protein-protein interaction network of YWHAB protein. Colored circle predicted functional partners of YWHAB. White circle means proteins that are regulated by the interaction of YWHAB with its functional partners. Different colors lines represent different evidence of connection between proteins (Dark green - neighborhood, red - gene fusion, blue - co-occurrence, black - co-expression, pink - experimental evidence, turquoise - database evidence, light green - evidence from text, violet - homology between the two proteins.

For purpose of gaining insights into the function and mechanism of the deregulated exomiRNA signature panel, we searched for putative miRNA-target interactions by using miRNet analysis software. The program was directed to identify target genes involving the greatest number of deregulated exomiRNAs, thus we introduced 6 exomiRNAS: exomiR-185-5p, -144-3p, -25-3p, -15a-5p, -451a and -107. No shared gene target was obtained with this combination. The unique combination combining the highest number of exomiRNAs was: exomiR-185-5p, -144-3p, -25-3p, -15a-5p, and -107. This combination was found to be involved in post-transcriptional regulation of 12 putative key genes (**Figure 3A**). Then, we used Reactome software to examine the signaling pathways where these 12 genes could be engaged. Interestingly, we observed that the 14-3-3 β gene (*YWHAB*), which encodes an adapter protein involved in regulating a broad spectrum of signaling pathways, was consistently identified in most of the deregulated pathways (**Figure 3B**, see **Supplementary Table S2**). Since YWHAB coded protein binds to a large number of proteins modulating its activity, we searched for its putative interacting network. STRING search results showed 20 putative proteins with interaction scores between 0.9 and 1 that the model considered true. The 20 proteins were mainly related with AKT signaling (AKT1, mTOR, TSC1, TSC2), MAPK kinase signaling (MAP2K1) and apoptosis (KRAS, NRAS, RAF1, BRAF, ARAF) among others. Interactions with adapter proteins from the same YWHA family were also observed (YWHAE, YWHAH, YWHAZ, YWHAG) (**Figure 3C**).

### Serum Biomarker Signature Can Identify Early RA

Spearman bivariate correlation analyses were used to measure the strength of the linear relationship between the study population and serum biomarkers (sTWEAK and sCD163), clinical parameters and exomiRNA expression levels (**Figure S1**). Considering only the significant associations, notably: a positive association between C-reactive protein levels and serum levels of ACPA (r=0.614, p ≤ 0.001), exomiR-185-5p (r=0.291, p=0.030), exomiR-15a-5p (r=0.329, p=0.013), exomiR-451a (r=0.481, p ≤ 0.001), exomiR-107 (r=0.278, p=0.038), serum sCD163 (r=0.291, p=0.03) and serum sTWEAK (r=0.275, p=0.04). ACPA showed a positive correlation with exomiR-185-5p (r=0.356, p=0.007), exomiR-25-3p (r=0.451, p ≤ 0.001), exomiR-451a (r=0.613, p ≤ 0.001), exomiR-107 (r=0.337, p=0.011), sCD163 (r=0.458, p ≤ 0.001) and serum levels of sTWEAK (r= 0.403, p=0.002).

**Figure 4:**
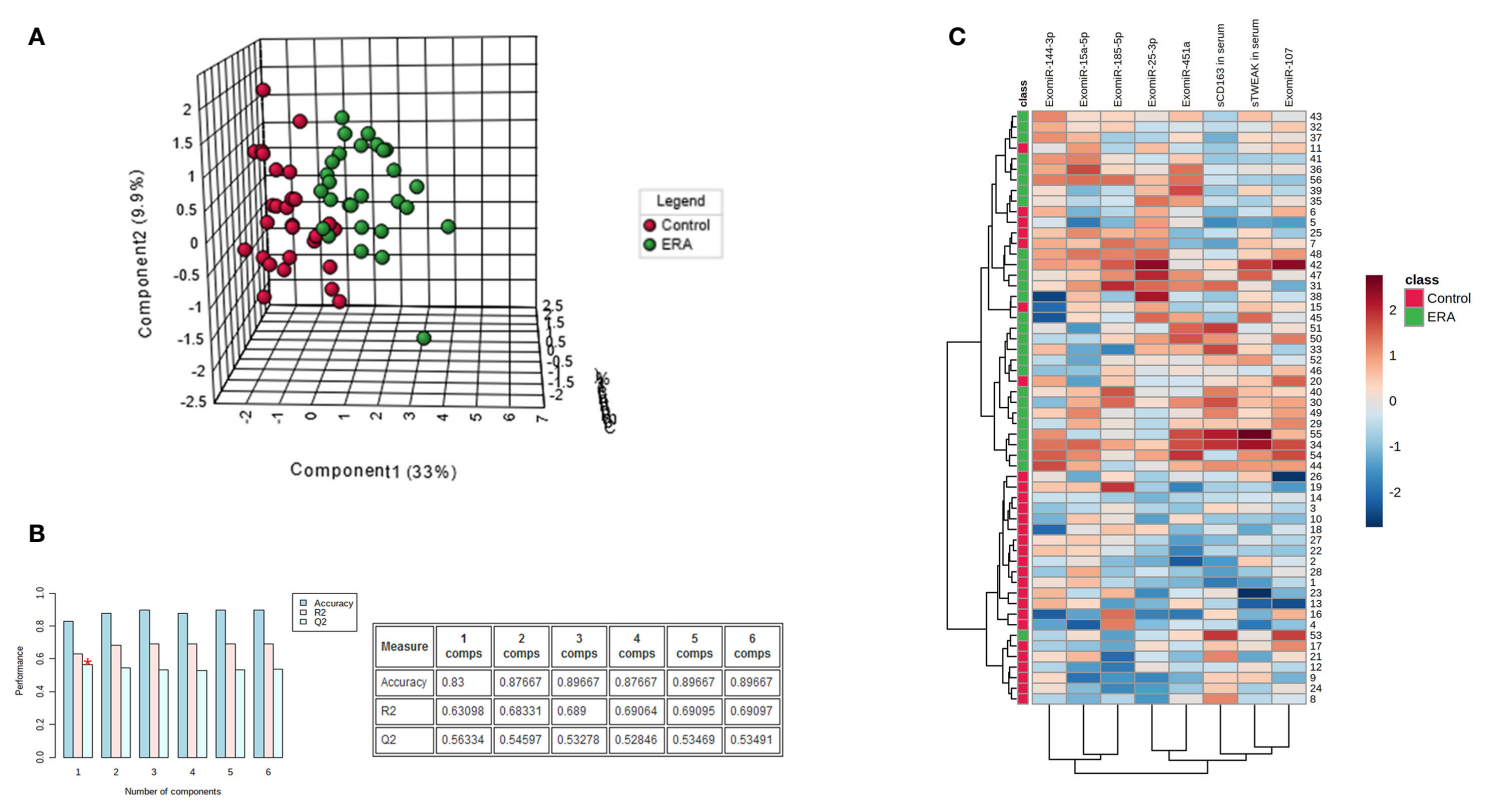
Serum biomarker signature panel identifies early RA. **(A)** PLS-DA analysis of the most representative exomiRNAs (exomiR-144-3p, exomiR-25-3p, exomiR-15a-5p, exomiR-451a, exomiR-107, exomiR-185-5p) and serum cytokine levels of sTWEAK and sCD163 demonstrate that two groups early RA and controls can be separated. **(B)** Model accuracy. **(C)** Heap map analysis of early RA samples and controls show discrimination between cohorts and different abundance of each specific biomarker.

To identify signature differences between the early RA and control groups, we developed a Partial Least Square Discriminant Analysis (PLS-DA) model using the following variables: exomiR-144-3p, exomiR-25-3p, exomiR-15a-5p, exomiR-451a, exomiR-107, exomiR-185-5p, sTWEAK and sCD163. PLS-DA analysis revealed that these variables could clearly discriminate between early RA and controls subjects (**Figure 4A**). The PLS-DA model over fitting, measured as the ratio of the Q2/R2 (R2—how well the model predicts the calibration of variables, and Q2—how well the model predicts ERA) was 0.88, indicating that the model fitted well (**Figure 4B**). A model is considered predictive when Q2/R2 ratio is greater than 0.5 (35).

We also performed a descriptive heat map analysis to visualize samples and variable clusterization. The results revealed two clearly differentiated cluster groups, fitting with the two studied cohorts (**Figure 4C**).

**Figure 5:**
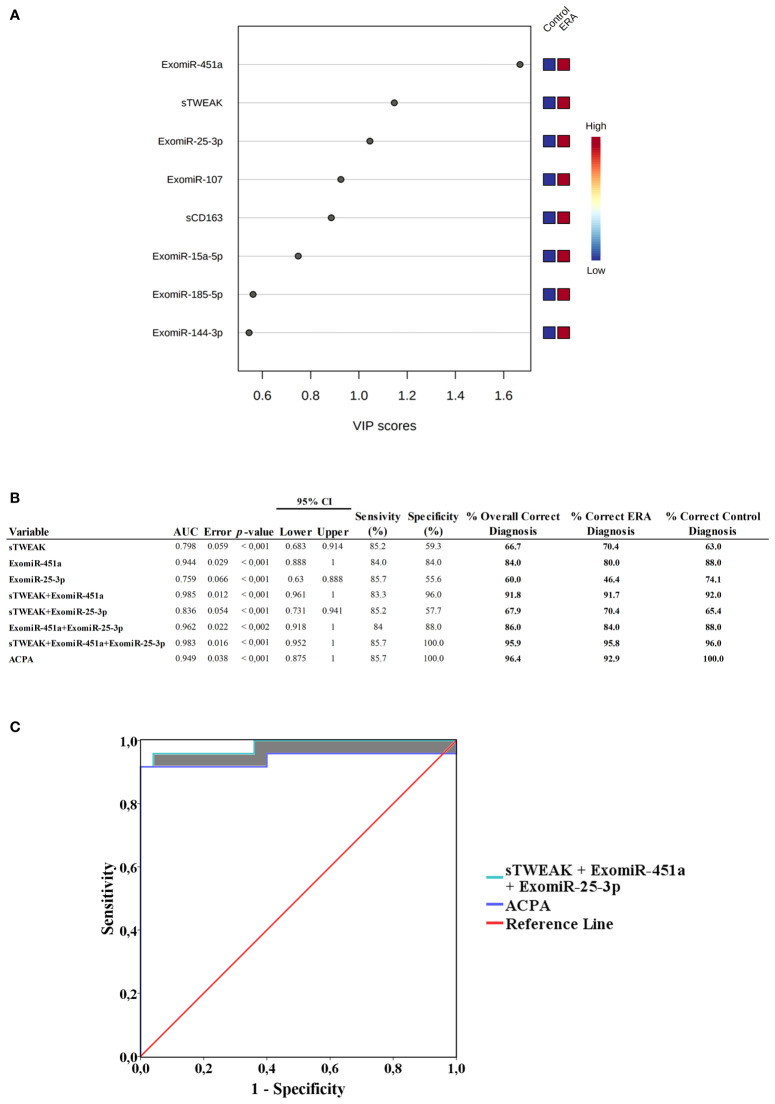
**(A)** Variable importance in projection (VIP) analysis, showing importance of each selected signature in discriminating between cohorts. Red squares indicate high expression and blue squares low expression **(B)** Receiver operating characteristic (ROC) curve values showing the predictive efficiency for distinguishing early RA from healthy controls. Percentage of correct diagnostic values was obtained by univariate models. AUC, area under the curve; 95% CI (confidence interval). **(C)** ROC area comparations. ROC area from ACPA was compared with ROC from sTWEAK + exomiR-451a + exomiR-25-3p. Shaded areas show ROC AUC differences.

The contribution of each of the above-mentioned selected variables to the observed group separation was evaluated by a variable importance in projection (VIP) model of multivariate analyses. Variables with VIP score ≥1 were considered important in the model for determining early RA disease. The model revealed that sTWEAK, exomiR-451a and exomiR-25-3p were the three most important variables in separating the early RA group of patients from the control group (**Figure 5A**).

To evaluate the usefulness of each VIP selected signature as potential diagnosis markers of early RA we performed logistic regression analysis and ROC curve analysis combining the following variables: sTWEAK and exomiR-451a and exomiR-25-3p. We also include ACPA levels for comparison. The different combinations are tabulated in **Figure 5B**. Results for the area under the curve (AUC) of each individual variable were: sTWEAK AUC=0.798; exomiR-451a AUC=0.944 and exomiR-25-3p AUC=0.759. We then used univariate regression model combining each potential biomarker to test which combination was more suitable for correct diagnosis of early RA. Notably, we observed that the presence of exomiR-451a plus sTWEAK outperformed the other individual variables alone or in combination. We found that the best panel to diagnose early RA comprised exomiR-451a, exomiR-25-3p and sTWEAK, which could correctly classify 95.6% of patients, with an AUC of 0.983 and with 100% specificity and 85.7% sensitivity (**Figure 5B**).

Binary logistic regression analysis showed that the panel composed of sTWEAK, exomiR-451a and exomiR-25-3p could correctly classify 96% of controls and 95.8% of patients with early RA, whereas ACPA alone could classify 100% of controls but only 92.9% of patients with early RA. Thus, the new biomarker signature outperformed the diagnostic power of the classical ACPA clinical biomarker by 2.9% (**Figure 5B**). Additionally, an increased AUC area was observed when comparing ACPA ROC area (AUC=0.949) with ROC area from the sTWEAK, exomiR-451a and exomiR-25-3p panel (AUC=0.983) (**Figure 5C**).

## Discussion

Early diagnosis of RA is crucial to prevent joint damage and improve quality of life. ACPA positivity is the most accepted disease predictor of RA (4, 36) and ACPA antibodies are detectable months-to-years prior to clinical manifestations (30). However, these antibodies are not exclusive to RA (37). This biomarker is not useful for patients with seronegative arthritis, which is considered in some studies to represent up to 37% of RA onset patients (38). False positive results for ACPA have been described in different autoimmune diseases (Sjogren’s syndrome, systemic lupus erythematosus), infectious diseases (hepatitis B virus, leishmaniasis, endocarditis) among others (39). Additionally, there is scientific evidence showing that when standard methods for early diagnosis of RA are not useful, nuclear magnetic resonance imaging may be superior in helping the diagnosis than ACPA antibodies determination (40).

Because early RA is difficult to diagnose due to the lack of effective biomarkers, the aim of the present study was to search for a diagnostic panel of soluble biomarkers that could aid clinicians in detecting preclinical RA.

Serum and synovial fluid have been very useful in providing information on the preclinical state of patients with RA. Accordingly, we focused our biomarker search on miRNAs packaged in serum-derived exosomes (exomiRNAS), as exomiRNAs have potential as diagnostic disease markers (22), and because they have the ability to regulate gene expression during disease onset, and are, therefore, good candidates for putative target detection (41).

We used a battery of tests to ensure that the isolated miRNAs originated from exosomes, including protein expression of exosomal markers, as recommended by the International Society for EVs (42), and size characterization, which revealed uniformity of size distribution.

Microarray analysis of serum exomiRNAs allowed us to identify 6 deregulated exomiRNAs (exomiR-144-3p, -451a, -25-3p, -15a-5p, -107 and -185-5p) in a cohort of patients with early RA. *In silico* analysis identified *YWHAB* gene as common target of 5/6 putative miRNA-regulated gene pathways. *YWHAB* encodes the 14-3-3 β protein, belonging to a family of seven YWHA proteins (43). YWHAB has been described as a negative regulator of osteogenesis (44) by inhibiting MAPK signaling (45, 46). *YHWAB* also interacts with some of its family members (*YWHAE, YWHAH, YWHAZ, YWHAG*) known to be involved in critical processes such apoptosis, cell cycle progression, autophagy, and glucose metabolism (43). Moreover, modeling analysis showed that YHWAB can bind to AKT1, a kinase of the PI3K/AKT signaling pathway involved in cartilage degradation and synovial inflammation (47–49), events related with RA pathogenesis (48, 50, 51). Included in this family is the intracellular protein 14-3-3 η (YWHAH), which can be externalized and citrullinated during the inflammatory process, and has been proposed to be of diagnostic utility in RA (7).

From the data here obtained one could hypothesize that reduced *YWHAB* gene expression could be anticipated in early RA patients due to exomiRNA-gene interactions and consequently, low YWHAB protein yield. Lower YHWAB binding-interaction with key-regulatory proteins involved in pathways such MAPK, AKT or apoptosis could be expected, thus potentiating these cascades. In this sense, our prediction models showed that YHWAB could interact with of BH3-domain proteins, known to be related to apoptosis (52). YHWAB has been reported to bind to BAD, a BCL-2 associated agonist of cell death promoting apoptosis (53). Therefore, if YHWAB protein levels decrease due to epigenetic miRNA control in early RA subjects, apoptosis can be expected to be inhibited, an event reported in RA pathogenesis (51, 54). However, our findings regarding YHWAB are *in silico*, thus further investigations are needed to corroborate its function in the pathology of RA.

Inflammation plays a prominent role in RA. This is reflected by the presence of elevated soluble inflammatory serum markers (8). The cytokine TWEAK is known to induce the production of other cytokines and has been reported to be involved in the pathogenesis of RA (55). We reported here for first time that serum levels of sTWEAK were higher in the early RA cohort than in matched controls. Likewise, CD163 a monocyte-macrophage soluble marker especially useful as a marker of immune activation (56) was detected elevated in our early RA cohort. Levels of sCD163 have been previously reported to predict radiographic progression in early RA patients but not for early detection since baseline sCD163 levels in early RA compare with healthy volunteers did not significantly change (19). Conversely, other authors, found sCD163 levels increased in early RA patients a time 0 of treatment when compared with healthy controls (56).

In the search for a useful panel of soluble serum biomarkers for the early RA diagnosis, we performed VIP analysis study as decision-making in choice from a set of several potential predictive RA variables. The results showed that the most predictive variables were exomiR-451a, exomiR-25-3p and sTWEAK in serum. ROC analysis indicated that the above three selected biomarkers could outperform the classical serum ACPA biomarker by 2.9%, improving the classification efficacy.

Bivariate correlation analysis revealed that ACPA levels, apart from positively correlating with CRP levels, a well-known marker to assess systemic inflammation (57), was found to be positively related with levels of sTWEAK and sCD163 in serum, indicating that these two biomarkers might be good preclinical inflammatory status indicators for early RA patients, corroborating previous findings on both biomarkers in established RA (15, 18).

To the best of our knowledge ours is the first study to isolate both exomiR-451a and exomiR-25-3p from serum exosomes. However, literature regarding these two miRNAs isolated freely in RA biofluids has been published. This is the case of miR-451 that has been proposed to play an important role in the pathogenesis of RA and has been found to be highly expressed in the articular synovial tissue and peripheral blood mononuclear cells from subjects at risk of developing RA (58). Moreover, miR-451 can inhibit the proliferation of synovial fibroblasts and also regulates p38 MAPK expression, resulting in cytokine downregulation (59). miR-25-3p has been recently considered as part of a epigenetic regulator panel of genes identify by bioinformatics analysis for the early diagnosis of RA (60). Functionally, miR-25-3p has been shown to suppress the apoptosis of rat chondrocytes and promote cell proliferation by targeting insulin-like growth factor-binding protein 7 (61).

We are aware that sample size may be one limitation in our study design. However, patients with these characteristics and naïve to treatment are difficult to recruit. Therefore, we believe that our data provides interesting evidence for scaling up future larger replication studies in early RA patients. Additionally, panel specificity needs to be tested in established RA and in other related inflammatory diseases.

In summary we have identified a set of deregulated exomiRNAs in early RA predicted to target *YHWAB*, which may have a relevant role in the development of RA. Moreover, we have obtained a panel combining exomiR-451a, exomiR-25-3p with serum levels of sTWEAK that can outperform the classical ACPA biomarker for correct diagnosis, improving the preclinical detection of early RA.

## Data Availability Statement

The original contributions presented in the study are included in the article/**Supplementary Material**. Further inquiries can be directed to the corresponding author.

## Ethics Statement

The studies involving human participants were reviewed and approved by Ethical Committee for Clinical research (CEIM) of the Pere Virgili Research Institute (Ref. CEIM: 047/2021). The patients/participants provided their written informed consent to participate in this study.

## Author Contributions

All authors were involved in drafting the article or revising it critically for important intellectual content, an all authors approved the final version to be published. SR-M, AA-C, and MRC had full access to all the data in the study and take responsibility for the integrity of the data and the accuracy of the data analysis. Study conception and design: SR-M, AA-C, RF-G, and MRC. Acquisition of data: SC-O and MP-E. Analysis and interpretation of data: AA-C, SR-M, RF-G, and MRC.

## Funding

This study was funded by the “Instituto de Salud Carlos III through projects PI17/00877 and PI20/00418 (co-founded by the European Regional Development Fund/European Social Found; “A way to make future”/” Investing in your future”), and by a grant from the “Societat Catalana de Reumatologia” awarded to SR-M.

## Conflict of Interest

The authors declare that the research was conducted in the absence of any commercial or financial relationships that could be construed as a potential conflict of interest.

## Publisher’s Note

All claims expressed in this article are solely those of the authors and do not necessarily represent those of their affiliated organizations, or those of the publisher, the editors and the reviewers. Any product that may be evaluated in this article, or claim that may be made by its manufacturer, is not guaranteed or endorsed by the publisher.
