# Supplementary material for: A Serum Biomarker Panel of exomiR-451a, exomiR-25-3p and Soluble TWEAK for Early Diagnosis of Rheumatoid Arthritis
Source: Front Immunol. 2021 Nov 15;12:790880. doi: 10.3389/fimmu.2021.790880 (PMC8636106; doi:10.3389/fimmu.2021.790880)
Supplement: Supplementary file 1 [file DataSheet_1.pdf]

Supplementary Material

Article:

"A serum biomarker panel of exomiR-451a, exomiR-25-3p and soluble TWEAK for early diagnosis of rheumatoid arthritis" By Samantha Rodríguez -Muguruza M.D. Ph. D, Antonio Altuna-Coy M.Sc., Sonia Castro-Oreiro M.D, Maria José Poveda-Elices M.D, Ramon Fontova-Garrofé M.D. Ph.D. and Matilde R. Chacón Ph.D.

Table S1

	**Control (n=4)**	**Early RA (n=4)**	
	**Mean ± SD**	**Mean ± SD**	***p -value***
**Age (Years)**	45.50 (38.75, 52.25)	42.00 (29.50, 50.00)	>0.999
**BMI (kg/m^2)**	29.82 (29.08, 32.10)	30.57 (26.54, 33.12)	>0.999
**Glucose (mmol/L)**	89.37 (77.75, 101.23)	87.50 (75.50, 102.50)	>0.999
**Uric Acid (mmol/L)**	4.10 (3.48, 5.66)	5.04 (3.55, 5.69)	>0.999
**Creatinine (µmol/L)**	0.72 (0.62, 0.79)	0.62 (0.44, 0.84)	>0.999
**Total Cholesterol (mmol/L)**	204.05 (140.25, 232.82)	164.70 (132.66, 198.69)	0.565
**HDL-Cholesterol (mmol/L)**	53.67 (47.49, 64.48)	46.20 (35.60, 61.08)	>0.999
**Triglycerides (mmol/L)**	86.29 (55.53, 115.71)	88.00 (76.50, 158.75)	0.812
**GGT (µkat/L)**	10.80 (8.85, 62.58)	23.50 (13.00, 34.75)	>0.999
**CRP (mg/L)**	0.40 (0.23, 1.97)	22.50 (11.00, 34.75)	<0.001
**ACPA (U/mL)**	1.18 (1.10, 1.54)	126.00 (88.90, 689.00)	0.021
**RF (UI/ml)**	ND	221.00 (183.50, 284.00)	-

Abbreviations: BMI, Body mass index; HDL, High-density lipoprotein; GGT, Gamma Glutamyltransferase; C-reactive protein, ACPA, anti-citrullinated protein/peptide antibody; RF, Rheumatoid Factor; ND, Not Determined

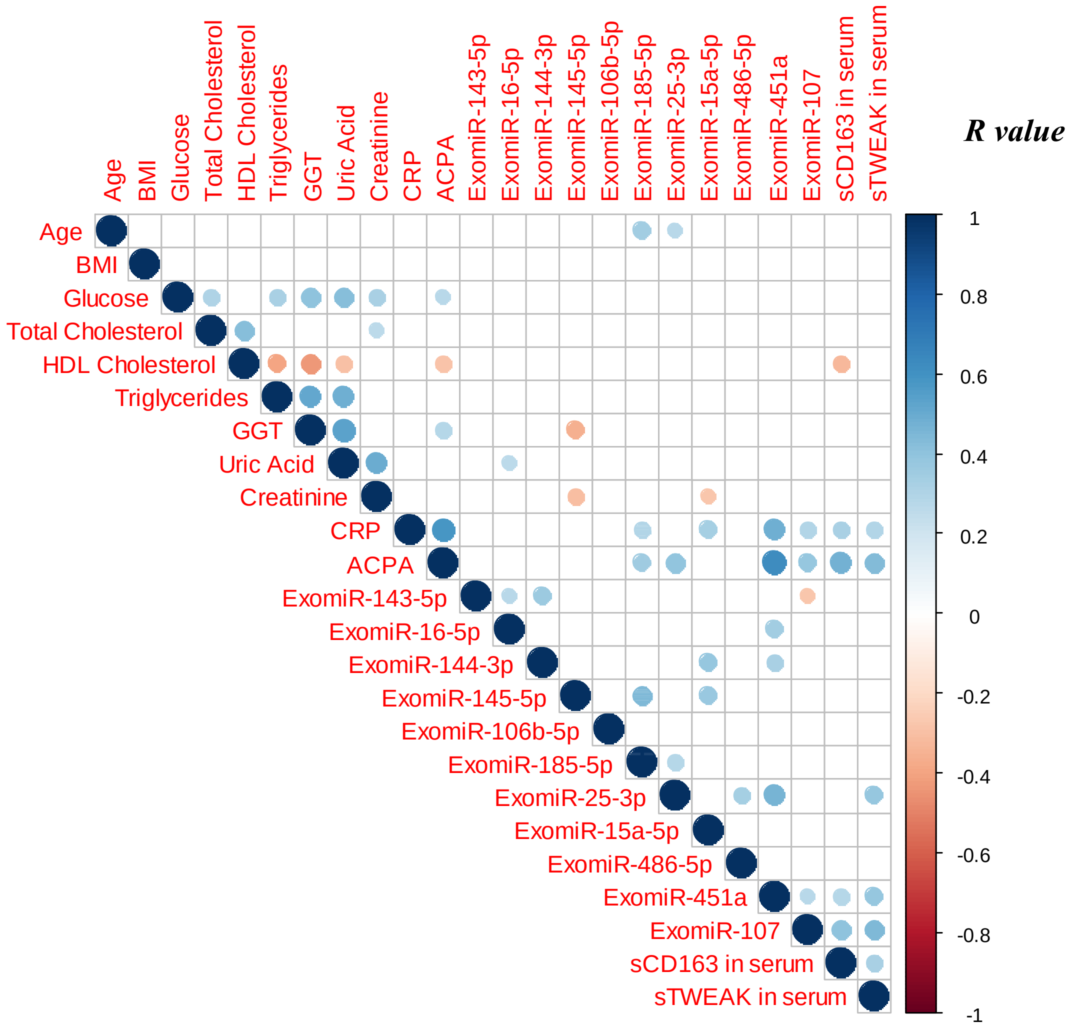

Figure S1. Spearman correlation matrix. Correlation map plotted using significance levels for Spearman test performed with relevant clinical and biomarker data from all studied patients. Positive correlations are displayed in grading-blue and negative correlations in grading-red color. Correlations with p-value ≥0.05 are considered as insignificant and are left blank. Color intensity and the size of the circle are proportional to the correlation coefficients. In the right side of the correlogram, the legend colour shows the correlation coefficients and the corresponding colors. Abbreviations: BMI, Body mass index; HDL, High-density lipoprotein; GGT, Gamma Glutamyltransferase; CRP, C-reactive protein, ACPA, anti-citrullinated protein/peptide antibody

Supplementary Western blot images

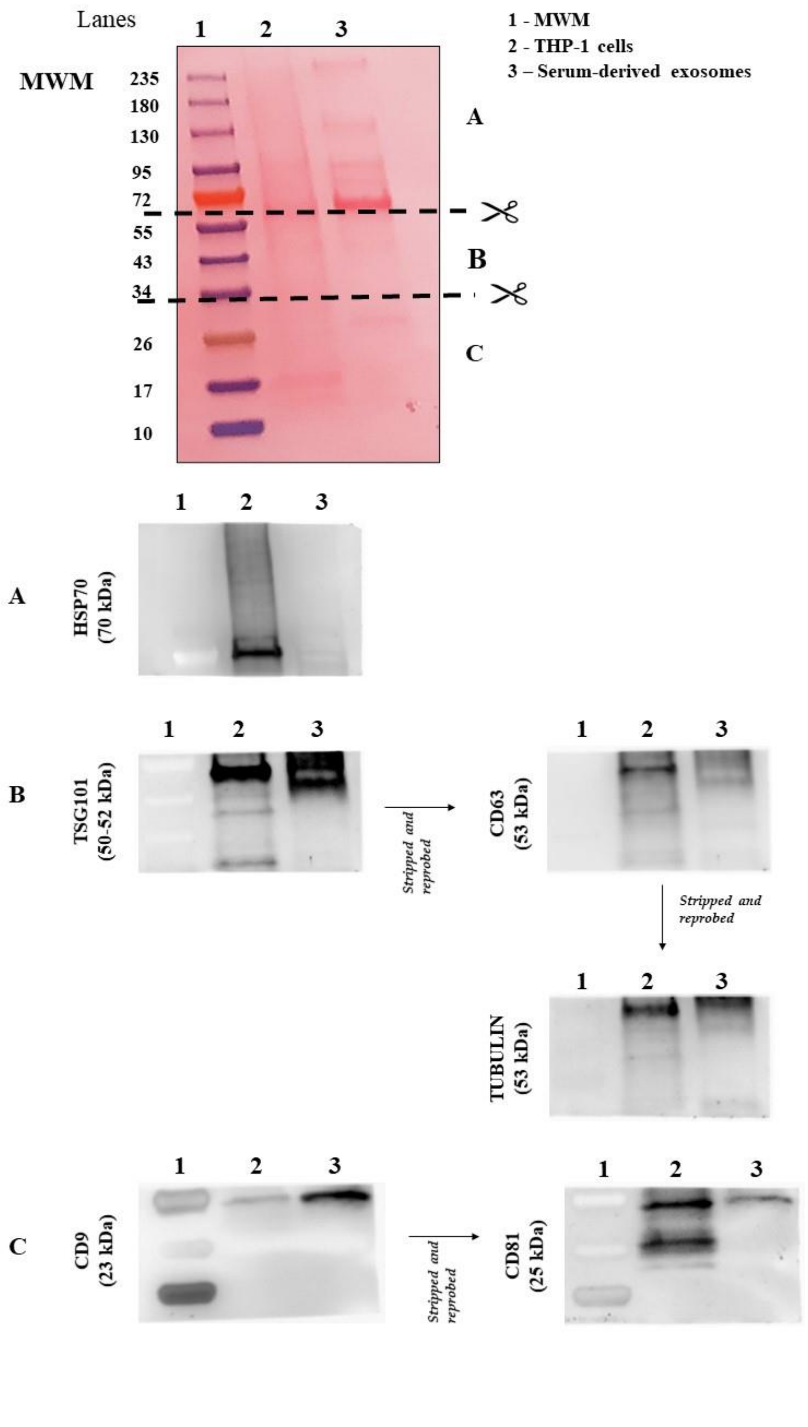

Complete WB results, referring to Figure 1B. Abbreviations: MWM, Molecular Weight Markers. Symbols: Scissor/dotted lines: position where membrane was cut and incubated with antibodies as indicated in each panel (A, B or C).

Table S2 - microRNA binding sites predicted for YWHAB by STarMir (https://sfold.wadsworth.org/cgi-bin/index.pl)

3' UTR Gene Target	miRNA name	LogitProb	Seed Start	Seed End	Seed Type
YWHAB	hsa-miR-144-3p	0.673	1171	1176	6mer
YWHAB	hsa-miR-144-3p	0.682	1266	1271	6mer
YWHAB	hsa-miR-107	0.714	1523	1529	7mer-m8
YWHAB	hsa-miR-107	0.657	1460	1465	offset-6mer
YWHAB	hsa-miR-107	0.569	1428	1434	7mer-m8
YWHAB	hsa-miR-107	0.550	1365	1370	offset-6mer
YWHAB	hsa-miR-15a-5p	0.564	1524	1529	offset-6mer
**CDS Gene Target**	**miRNA name**	**LogitProb**	**Seed Start**	**Seed End**	**Seed Type**
YWHAB	hsa-miR-25-3p	0.511	568	573	6mer
YWHAB	hsa-miR-25-3p	0.511	473	478	6mer
YWHAB	hsa-miR-185-5p	0.662	299	304	7mer-A1
YWHAB	hsa-miR-185-5p	0.558	742	747	6mer

Heading table explanation: 3'UTR, three prime untranslated regions; **CDS**, Coding Sequence; **LogitProb**, Probability of the site being a miRNA binding site as predicted by a quadratic logistic model**; Seed Start**, Start position of the target sub-region complementary to the miRNA seed; **Seed End**, End position of the target sub-region complementary to the miRNA seed; **Seed Type**, types of miRNA target sites: **6mer**, an exact match to positions 2-7 of the seed mature miRNA; **7mer-m8**, An exact match to positions 2-8 of the mature miRNA (the seed + position 8); **offset-6mer**, An exact match to positions 3-8 of the mature miRNA (the seed + position 8); **7mer-A1**, An exact match to positions 2-7 of the mature miRNA (the seed) followed by an 'A'.
